# Protective Effect of Hand-Washing and Good Hygienic Habits Against Seasonal Influenza: A Case-Control Study

**DOI:** 10.1097/MD.0000000000003046

**Published:** 2016-03-18

**Authors:** Mingbin Liu, Jianming Ou, Lijie Zhang, Xiaona Shen, Rongtao Hong, Huilai Ma, Bao-Ping Zhu, Robert E. Fontaine

**Affiliations:** From the Department of Infectious Diseases (ML), Nanchang Center for Disease Control and Prevention, Nanchang; Chinese Field Epidemiology Training Program (ML, LZ, HM), Chinese Center for Disease Control and Prevention, Beijing; Office of Public Health Preparedness and Response (JO, XS, RH), Fujian Center for Disease Control and Prevention, Fuzhou, China; and Centers for Disease Control and Prevention (B-PZ, REF), Atlanta, GA.

## Abstract

Previous observational studies have reported protective effects of hand-washing in reducing upper respiratory infections, little is known about the associations between hand-washing and good hygienic habits and seasonal influenza infection. We conducted a case-control study to test whether the risk of influenza transmission associated with self-reported hand-washing and unhealthy hygienic habits among residents in Fujian Province, southeastern China.

Laboratory confirmed seasonal influenza cases were consecutively included in the study as case-patients (n = 100). For each case, we selected 1 control person matched for age and city of residence. Telephone interview was used to collect information on hand-washing and hygienic habits. The associations were analyzed using conditional logistic regression.

Compared with the poorest hand-washing score of 0 to 3, odds ratios of influenza infection decreased progressively from 0.26 to 0.029 as hand-washing score increased from 4 to the maximum of 9 (*P* < 0.001). Compared with the poorest hygienic habit score of 0 to 2, odds ratios of influenza infection decreased from 0.10 to 0.015 with improving score of hygienic habits (*P* < 0.001). Independent protective factors against influenza infection included good hygienic habits, higher hand-washing score, providing soap or hand cleaner beside the hand-washing basin, and receiving influenza vaccine.

Regular hand-washing and good hygienic habits were associated with a reduced risk of influenza infection. These findings support the general recommendation for nonpharmaceutical interventions against influenza.

## INTRODUCTION

Influenza is a highly transmissible RNA virus that causes seasonal epidemics and sometimes risk for pandemic, which are characterized by increased health care use and significant disease burden. Influenza is transmitted via 3 modes: contact, droplet, and airborne.^1^ Influenza viruses can stay viable on nonporous surfaces at room temperature for up to 3 days.^2,3^ Accordingly, the virus can contaminate hands that contact these surfaces. The virus could also be transferred from the hands of an infected person to the hands of a susceptible person. The actual degree to which this happens and the effectiveness of hand-washing at mitigating transmission via the hands are important for informing recommendations on controlling influenza during seasonal and pandemic situations. Currently, vaccination is the principal preventive measure to control influenza, but the desired protective effect is not always achieved. Certain high-risk individuals may have poorly responsive immune systems. Sometimes the antigenic match between the vaccine and the circulating influenza virus strain is suboptimal. An antigenic shift resulting in a new pandemic strain can render existing vaccine composition ineffective.^4–6^ Alternatively, some clinically used neuraminidase inhibitors (NIs), such as oseltamivir and zanamivir, could be useful for the pre-/postexposure prophylaxis for seasonal or pandemic influenza. Overuse of the NIs may select for resistant influenza strains, which might make NIs unusable in the future. In these circumstances, nonpharmaceutical interventions, including hygiene and social distancing, are recommended by the World Health Organization and the Centers for Disease Control and Prevention (CDC) to prevent the spread of influenza.^7^

The protective effect of hand-washing in reducing upper respiratory infections has been shown with randomized controlled trials.^8–11^ Most of the studies used symptoms as an outcome, and none of these involved confirmed influenza.^8–11^ The rapid spread of Influenza A (H1N1) pdm09 around the world emphasized the urgency to address the effectiveness of hand hygiene against influenza transmission before the pandemic arrived in China.

We carried out a case-control study based on laboratory confirmed seasonal influenza cases to test whether the risk of influenza transmission is associated with self-reported hand-washing and unhealthy hygienic habits (picking nose, touching mouth, and rubbing eyes), seasonal influenza vaccination, and environmental factors among residents in 5 cities of Fujian Province, southeastern China.

## METHODS

### Ethics Statement

This study was approved by the Institutional Review Board of Nanchang Center for Disease Control and Prevention, China. The institutional review board stated that written consents from patients were not required for this study because the identification numbers and personal information about participants were not included in the secondary files. All participants provided their verbal, informed consent.

### Sample Collection and Laboratory Test

Nasopharyngeal swabs were collected from patients with influenza-like illness (ILI) through 11 sentinel surveillance sites of the Chinese National Influenza Surveillance System in 5 cities in Fujian Province. Patient selection, collection of samples, and laboratory methods of this system have been described in detail elsewhere.^12,13^ Briefly, patients presenting to hospital outpatient departments with ILI were enlisted to provide a nasopharyngeal sample for influenza virus culture. Nasopharyngeal specimens were collected using a sterile swab, put into viral transport medium (Qiagen, Germany), and kept at 2 °C to 4 °C. Before the end of the day of collection, samples were sent to the nearest influenza surveillance laboratory using a cool box with at least 2 icepacks. Samples were eluted and cryopreserved at −70 °C immediately after receipt. All clinical specimens were cultured on Madin–Darby canine kidney cells with exogenous trypsin (Qiagen, Germany) (2 μg/mL) added. Influenza virus isolates were subtyped by a real-time reverse transcriptase polymerase chain reaction using Qiagen OneStep RT-PCR Kit (Qiagen, Germany). All laboratory tests were completed within 2 to 3 days after the samples were received.

## PARTICIPANTS

ILI patients with positive influenza testing were immediately reported to a study coordinator when the diagnosis was made. Patients aged ≥3 years were consecutively included in the study as case-patients from March 1 to June 30, 2009.

Control persons were residents of the same city as case-patients. We excluded from control persons who had symptoms of influenza^13^ (fever ≥38 °C, and cough or sore throat) or pneumonia or who had been treated with antivirals during the 7 days before the interview. We selected 1 control for each case, matched by age (±1 year) and city of residence. Control persons were obtained by using random digit dialing, using telephone number lists of the county as the case-patients. Before beginning the interview we first determined if the potential control persons lived in the same community as the matched case-patients.

### Telephone Interview

We used a standardized, structured questionnaire that covered demographic factors, vaccination status, environmental parameters, and influenza transmission-related behaviors. Information on exposure to these risk factors was collected for 1 week up to the reference date–that is, the date of onset for case-patients and the date of interview for control persons.

We conducted telephone interviews between 7:00 and 9:00 PM on the day we received the case-patients information. Each interview lasted ∼30 minutes. We first told participants that the phone call involved “issues about personal health”. After obtaining verbal consent to proceed, we explained that the focus was influenza. Respondents were required to be 18 years or older and to speak Putonghua (Mandarin Chinese). For those participants aged <18 years, their guardians, including parents or grandparents, were asked to help answer the questionnaire.

### Personal Variables

Trained staff checked the data to ensure quality, completeness, and validity. Personal information consisted of sex, age, address, working status, monthly household income, number in household, educational level, vaccination status, and the presence of any ILI cases among acquaintances, colleagues, or classmates. For both cases and controls we asked about influenza vaccination for the 2008 to 2009 winter influenza season. To improve accuracy of response on vaccination status we asked about date and place of vaccination and type of vaccine.

### Environmental Parameters

Participants were asked 7 questions about their households, workplace, or classrooms. Two related to duration of ventilation and 2 related to the area of enclosed environment in which respondents usually stayed within the past 7 days. One covered the number of people working or studying in the same office or classroom. Two covered the hand-washing facilities provided by the employer or school.

## BEHAVIORS

Thirteen items were used to assess key activities or behaviors related to influenza virus transmission, these questions were phrased as “Over the past seven days (for cases we added ‘before your onset of flu’), I have. . . ”. Of all 13 questions, six were related to crowded places that the government had advised avoiding to prevent influenza infection, permitted responses for each question were “yes” or “no”; four were related to recommended activities for influenza prevention, namely increased washing hands with soap and water before eating, after using toilet, and after returning home from community activities, response options were “never or rarely” (scored as 0), “sometimes” (1), “often” (2), or “every time” (3); three were used to assess the frequency that participants touched their eyes, mouth, and nose by hands, with response options ranging from “often” (scored as 0), “sometimes” (1), “seldom” (2), to “never” (3).

### Statistical Analysis

To evaluate the effect of hand-washing and poor hygienic habits on influenza transmission, we represented the relative frequency of hand-washing with the sum of the responses of the 4 hand-washing situations. Similarly, we summed the responses of the poor hygienic habits to create a hygienic habit score. We also used the sum of the calculated total score for crowding places experienced, excluding hospital or clinic exposures. These were considered as a separate independent risk factor for infection. We calculated the average per capita area of household and office or classroom by dividing the estimated living or working area (m^2^) by the number of household members, workmates, colleagues, or classmates. To examine characteristics associated with influenza transmission, we calculated proportions for categorical variables, and medians and interquartile ranges for continuous variables. Dummy variables were created for hand-washing, hygienic habits, crowded places scores, and other variables.

We used conditional logistic regression models to estimate odds ratios (ORs) and their corresponding 95% confidence intervals (95% CIs) for the bivariable and multivariable analysis. We first computed crude (unadjusted) ORs associated with each exposure group in bivariable analysis. To construct final multivariable model and adjust for potential confounders, we used the backward stepwise procedure for variables significant at the 5% level in the bivariable analysis. We retained only variables with significance levels of *P* < 0.10 in the final model. We tested for trend by entering the categorical variables as continuous parameters in the models. A few variables had missing values. For bivariable analysis we excluded the case-control pairs with a missing value from the analysis. We used STATA version 10 (Stata Corp, USA) for analysis.

## RESULTS

### Study Population

During the study period, March 1, 2009, to June 30, 2009, the influenza surveillance system identified 173 laboratory confirmed influenza infections. Of 154 case-patients that met inclusion criteria, 54 had given an incorrect telephone number, initially refused to participate, or withdrew during the interview; 100 participated in the case-control study (participation rate: 65%). Virus types included A H1 (47%), A H3 (29%), B Victoria (15%), and Yamagata (9%). None were A (H1N1)pdm09. We contacted 531 potential participants during recruitment of controls. Of these, 180 did not meet the inclusion criteria. Of the remaining 351, 162 (46%) declined to participate and 89 (25%) withdrew during the interview leaving 100 completed control interviews for this study (participation rate: 28%). Median age was 10 years (interquartile range 5.5 to 25) for cases and 10 years (5 to 25) for controls. Fifty-nine percent of the case-patients and 52% of control persons were male.

### Environmental Risk Factors for Influenza Infection

Among the environmental factors the strongest effect (OR = 0.13) was with availability of soap or hand cleaner at the hand-washing basins of schools or workplaces (Table 1). Note that the availability of hand-washing basins alone was nearly identical between case-patients (75%) and control persons (82%). Weaker effects were observed with crowding at the home, workplace, or school. Only 17% of both case-patients and control persons knew a person at work or school who had acute respiratory illness (ARI) 7 days before onset of their own ILI (for cases) or 7 days before the interview (for controls).

**TABLE 1:**
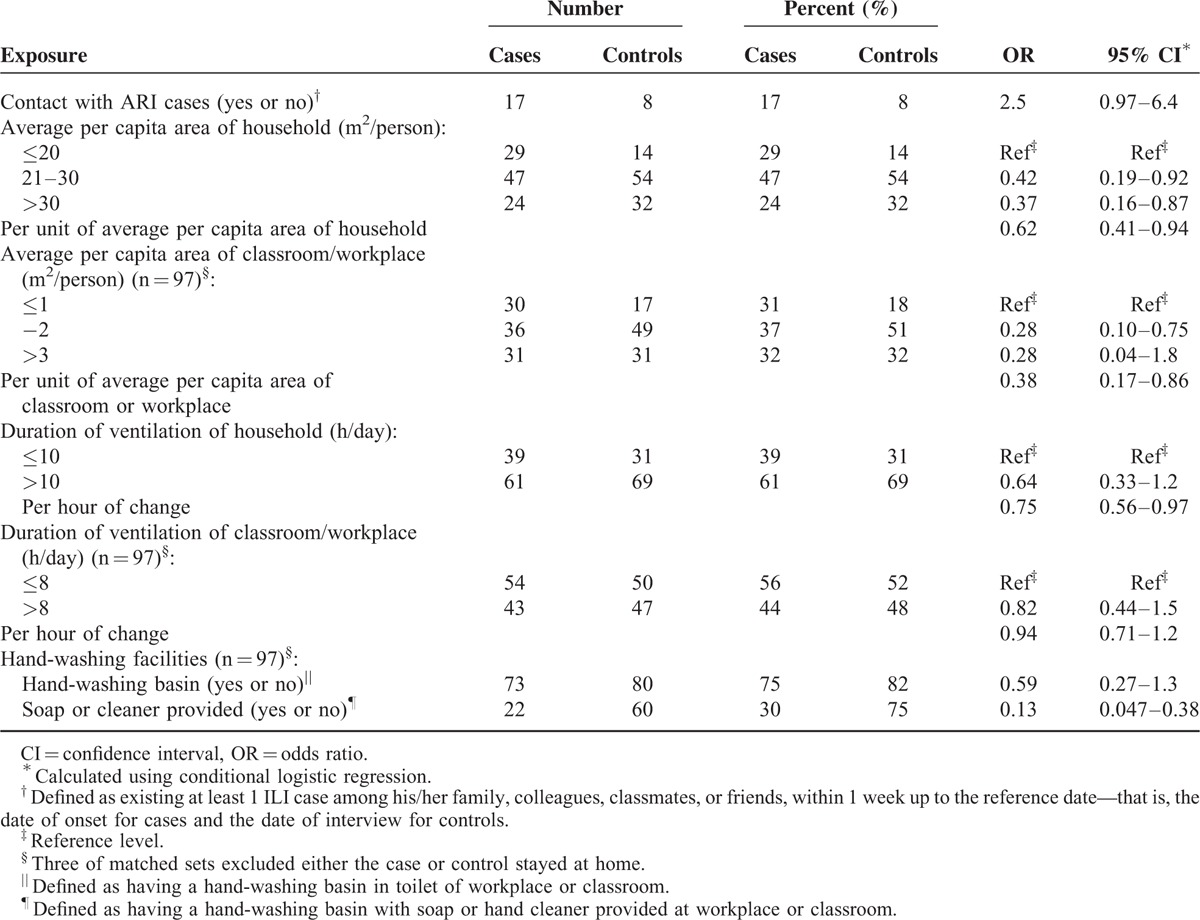
Personal and Environmental Exposures of 100 Influenza Cases and 100 Matched Controls, Fujian Province, China, March to June 2009

### Behavioral Risk and Protective Factors for Influenza Infection

In the univariate analysis the odds of influenza infection decreased with frequency of hand washing in all 4 situations showing a maximum protective effect (1-OR) of ∼78% to 88% depending on the hand-washing situation (Table 2). Using soap or hand cleaner when washing hands was also associated with protective effect of 78%. Low reported frequency of putting ones hands to the eyes, nose, or mouth showed a protective effect of 86% to 88% for each site.

**TABLE 2:**
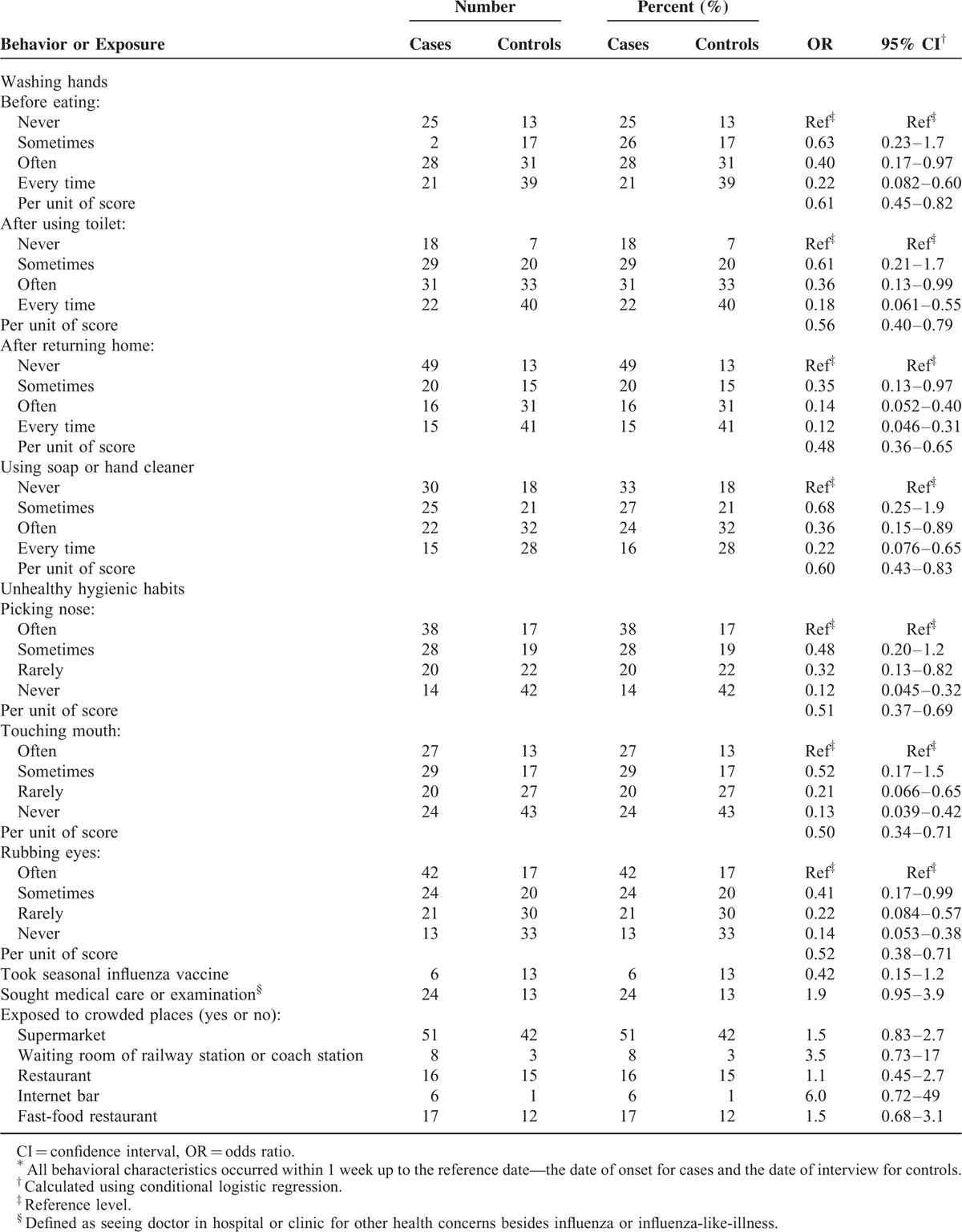
Behavioral Characteristics Associated With Influenza^∗^, Fujian Province, China, March to June 2009

Using a summary score for all hand-washing situations and for all hand-to-face behaviors, we observed an even stronger protective effect (Table 3). As the hand-washing score increased, the protective effect towards confirmed influenza also increased steadily from 74% to 97% compared to those with the poorest hand-washing (score 0–3). Similarly, the protective effect of reduced hand-to-face touching increased from 90% to 98%. A statistically significant association with the frequency of visits to crowded public places appeared only at the highest exposure frequency and involved 9% of case-patients and 3% of control persons.

**TABLE 3:**
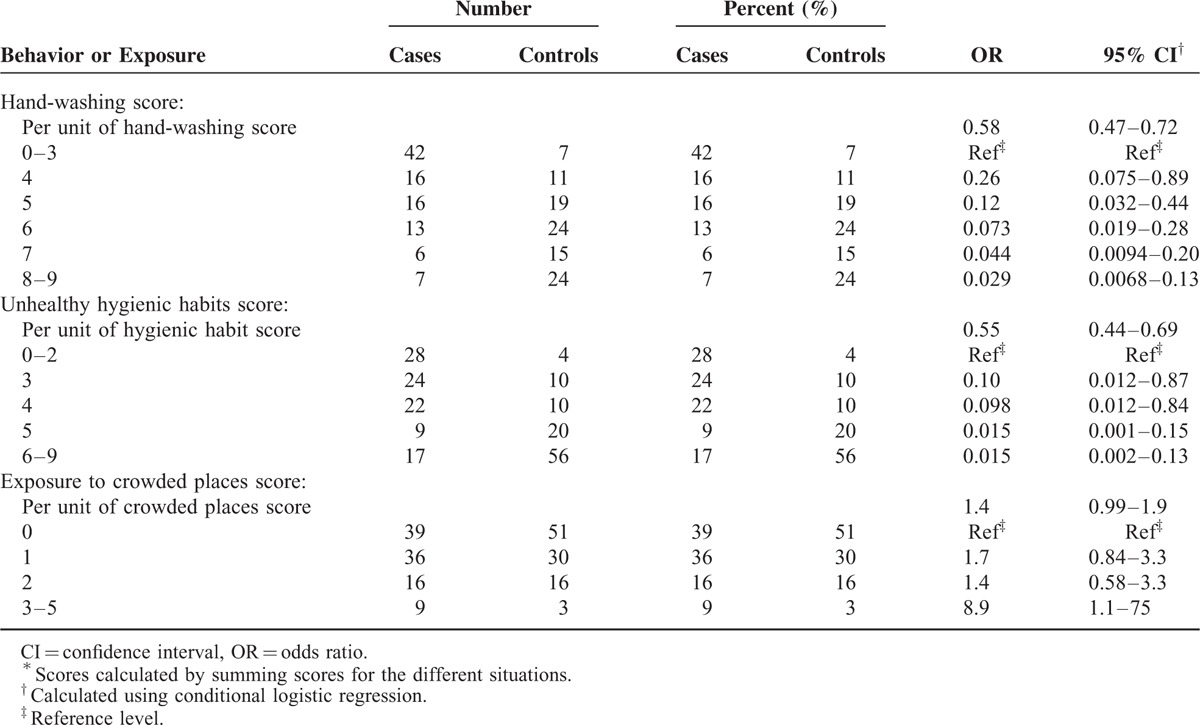
Hand-Washing, Unhealthy Hygienic Habits, and Exposure to Crowded Places Scores^∗^ and Risk of Influenza, Fujian Province, China, March to June 2009

In the multivariable analysis, both hand-washing and infrequent hand-to-face touching had strong protective effects against influenza infection of >95% for the top 3 of the 6 levels of hand-washing and the top 2 of the 5 levels of reduced hand-to-face touching (Table 4). Frequency of using soap for hand-washing, although statistically significant in the bivariable analysis, was highly correlated with frequency of hand-washing (Spearman's correlation coefficient 0.45, *P* < 0.0001) and was not associated with a protective effect. Having soap or hand cleaner available at the hand-washing basins at school or the workplace had a protective effect of 85%. Influenza vaccine for the 2008 to 2009 season had a vaccine effectiveness (1-OR) of 94%, but vaccine coverage was 6% among case-patients and 13% among control persons. Although we included 5 environmental variables reflecting crowding, indoor ventilation, contact with person with ARI, and visiting a medical clinic, none achieved the inclusion limit (*P* < 0.10) for the final multivariable model.

**TABLE 4:**
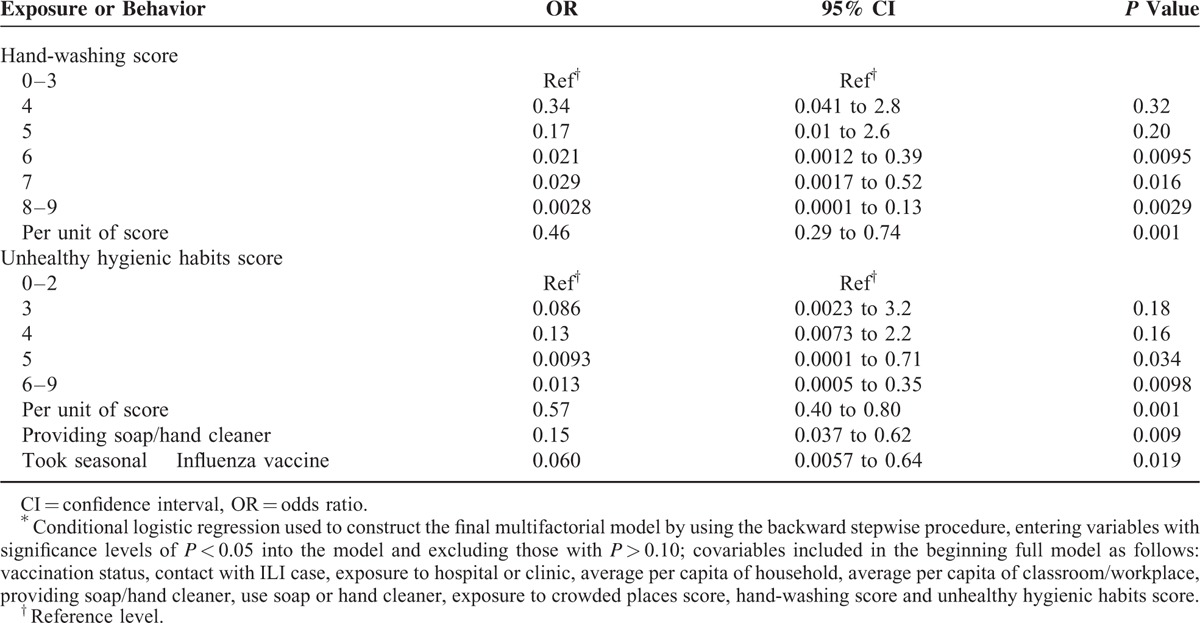
Independent Associations With Influenza Infection, by Multivariable Analysis^∗^, Fujian Province, China, March to June 2009

## DISCUSSION

Our study found a substantially lower risk of community-acquired influenza infection associated with self-reported personal behaviors including frequent hand-washing, infrequent touching the eyes, nose, or mouth with ones hands, and receiving seasonal influenza vaccine. All influenza cases included in our study were laboratory confirmed, thus the results apply specifically to influenza rather than to a mix of infectious agents causing ILI or ARI with unknown variability in transmissibility. Furthermore, this study was done among otherwise healthy school-age children and younger working adults; these results are best applied to prevention of community transmission of influenza among healthy active populations. This study was also done during seasonal influenza transmission just before pandemic H1N1 struck China. Under pandemic situations, social distancing measures to reduce crowding, to isolate ILI patients, and to close schools or public places would be far more intense. Accordingly, our results should be extrapolated carefully to these extraordinary situations.

A protective effect of self-reported hand-washing was seen in 2 other studies,^14,15^ of which 1 had a limited scope but selected from the general population.^14^ It found 79% protection with frequent hand-washing, but reported on no other risk or protective factors. The other, more comprehensive assessment in Spain,^15^ selected cases and controls from 2 strata, hospitalized and outpatients. Thus, it reflected influenza risk in a higher risk population. Similar to our study in Fujian, it revealed a dose–response effect with hand-washing. In Fujian we found more extreme protection at the better levels of hand-washing, >95% compared to 40% in Spain. Two factors could explain this difference. Fujian has only emerged economically in the past 20 years and hand-washing among the population may not have reached the overall level as Spain. Accordingly, the reference level of poor hand-washing for Fujian could be worse than for Spain. Second, our questions and analysis may have extended the measured range of hand-washing relative to Spain.

Interventional studies of hand-washing and influenza show lower protective effects than these case-control studies. The strongest effect, 50% against confirmed A (H1N1) pdm09 influenza, appeared in a broad school-based intervention in Egypt.^16^ Another school-based intervention study in the U.S. showed a protective effect, 19%, only for influenza A virus.^17^ A college dormitory intervention study showed similar protective effect of 43% from hand hygiene plus facemask. A household intervention study yielded no effect (−15%).^18^ Two studies intervened against secondary transmission in households with hand-washing and/or wearing facemasks.^19,20^ In these 2 studies, participants with an influenza-positive test and their household contacts were randomly assigned to 1 of 3 study groups: control, hand-washing, or hand-washing plus surgical face masks group. However, both hand-washing and face mask use showed weak, sometimes statistically nonsignificant, protective effects from 41% to −20%. Interventions were not applied until after ILI onset of the primary case, potentially accounting for the weak, observed protective effect. Compared with the open community, the confines of a home probably have greater potential for transmission by aerosols and expelled droplets. This would compete with transmission prevention through hand-hygiene and reduce its observed protective effect. The intervention studies may improve the hand-washing behavior from its status when the intervention was initiated to a point further along the scale from worst to best. They cannot replicate the existing breadth of hand-washing quality. By looking at the entire community, the case-control approach probably covered a relatively broad spectrum of hand-washing thus in part accounting for the 99% protective effect of the best compared to the poorest levels of hand-washing.

Influenza virus may be spread indirectly via fomites, by aerosols in confined spaces, and by direct transfer of expelled droplets.^1,21^ Our findings suggest that much of the community transmission was indirect from fomites via the unwashed hands to the mouth, nose, or eyes. Proximity to another person with ARI was infrequent. Visits to crowded buildings where unrecognized direct contact or proximity to an person with ARI could go unnoticed was associated with influenza only in the highest exposure category, among 9% of cases and 3% of controls, and only in the unadjusted analysis. Although we did not ask about touching potentially contaminated surfaces in our study, another case-control study did show an association with not washing hands after touching these surfaces.^15^ Influenza virus when deposited on nonporous surfaces can remain viable for 48 h or more.^2^ Influenza virus has been detected on surfaces in 23% of daycare centers during influenza transmission seasons and in 50% of homes with an influenza case.^22,23^ Experimental evidence imposes a caveat to the hand-washing protection. Influenza virus loses infectivity within 30 minutes when deposited on hands directly or from fomites,^2,22^ implying that hands are self-sterilizing. To have a protective effect, hand-washing would need to be done within minutes after touching a contaminated object. These experiments were done without using mucus or natural respiratory secretions as a carrier for the virus. Other experiments show that these carriers greatly prolong influenza virus viability on fomites.^3^ Replicating this finding on hands would further substantiate and refine the evidence on the protective effect of hand-washing on fomite transmission of influenza.

Avoiding crowding is recommended to reduce spread of pandemic influenza within populations. Reported studies of the effects of reducing crowding and implementing other nonpharmaceutical interventions on the incidence of respiratory illnesses were reviewed by Lee,^24^ who concluded that reducing crowding and using related interventions (provision of adequate living space, air dilution/ventilation) may be beneficial but deserve further evaluation. We found no statistically significant risk of influenza associated with either exposure to crowded places or duration of air ventilation, except the slight protective effect of average per capita area of household.

In conclusion, our results showed a reduction in risk of influenza associated with regular hand-washing and low frequency of hand-to-face contact. Taken together, these behaviors may account for a substantial proportion of cases of influenza in this study population. Informing the public about the benefits of hand-washing and the hazard of hand-to-face contact along with a strong influenza vaccination effort may be helpful in reducing the incidence of influenza.

## References

[1] Weber TP Stilianakis NI Inactivation of influenza A viruses in the environment and modes of transmission: a critical review. *J Infect* 2008; 57:361–373. 18848358 10.1016/j.jinf.2008.08.013 PMC7112701

[2] Bean B Moore BM Sterner B Survival of influenza viruses on environmental surfaces. *J Infect Dis* 1982; 146:47–51. 6282993 10.1093/infdis/146.1.47

[3] Thomas Y Vogel G Wunderli W Survival of influenza virus on banknotes. *Appl Environ Microbiol* 2008; 74:3002–3007. 18359825 10.1128/AEM.00076-08 PMC2394922

[4] Nichol KL Nordin JD Nelson DB Effectiveness of influenza vaccine in the community-dwelling elderly. *N Engl J Med* 2007; 357:1373–1381. 17914038 10.1056/NEJMoa070844

[5] Belongia EA Kieke BA Donahue JG Effectiveness of inactivated influenza vaccines varied substantially with antigenic match from the 2004–2005 season to the 2006–2007 season. *J Infect Dis* 2009; 199:159–167. 19086915 10.1086/595861

[6] Herrera GA Iwane MK Cortese M Influenza vaccine effectiveness among 50–64-year-old persons during a season of poor antigenic match between vaccine and circulating influenza virus strains: Colorado, United States, 2003–2004. *Vaccine* 2007; 25:154–160. 17064823 10.1016/j.vaccine.2006.05.129

[7] Bell DM Non-pharmaceutical interventions for pandemic influenza, national and community measures. *Emerg Infect Dis* 2006; 12:88–94. 16494723 10.3201/eid1201.051371 PMC3291415

[8] Larson EL Lin SX Gomez-Pichardo C Effect of antibacterial home cleaning and handwashing products on infectious disease symptoms: a randomized, double-blind trial. *Ann Intern Med* 2004; 140:321–329. 14996673 10.7326/0003-4819-140-5-200403020-00007 PMC2082058

[9] Luby SP Agboatwalla M Feikin DR Effect of handwashing on child health: a randomised controlled trial. *Lancet* 2005; 366:225–233. 16023513 10.1016/S0140-6736(05)66912-7

[10] Ponka A Poussa T Laosmaa M The effect of enhanced hygiene practices on absences due to infectious diseases among children in day care centers in Helsinki. *Infection* 2004; 32:2–7. 15007735 10.1007/s15010-004-3036-x

[11] Sandora TJ Taveras EM Shih MC A randomized, controlled trial of a multifaceted intervention including alcohol- based hand sanitizer and hand-hygiene education to reduce illness transmission in the home. *Pediatrics* 2005; 116:587–594. 16140697 10.1542/peds.2005-0199

[12] Cowling BJ Fung RO Cheng CK Preliminary findings of a randomized trial of non-pharmaceutical interventions to prevent influenza transmission in households. *PLoS One* 2008; 3:e2101. 18461182 10.1371/journal.pone.0002101 PMC2364646

[13] World Health Organization. A practical guide to harmonizing virological and epidemiological influenza surveillance. Available at: http://www.wpro.who.int/emerging_diseases/documents/docs/GuideToHarmonizingInfluenzaSurveillancerevised2302.pdf?ua=1 Accessed April 14, 2015.

[14] Liu WT Pang XH Deng Y A case-control study of the transmission of pandemic influenza A (H1N1) virus in families. *Zhonghua Jie He He Hu Xi Za Zhi* 2011; 34:509–514.[in Chinese]. 22041776

[15] Godoy P Castilla J Delgado-Rodríguez M Effectiveness of hand hygiene and provision of information in preventing influenza cases requiring hospitalization. *Prev Med* 2012; 54:434–439. 22548868 10.1016/j.ypmed.2012.04.009 PMC7119305

[16] Talaat M Afifi S Dueger E Effects of hand hygiene campaigns on incidence of laboratory-confirmed influenza and absenteeism in schoolchildren, Cairo, Egypt. *Emerg Infect Dis* 2011; 17:619–625. 21470450 10.3201/eid1704.101353 PMC3377412

[17] Stebbins S Cummings DA Stark JH Reduction in the incidence of influenza A but not influenza B associated with use of hand sanitizer and cough hygiene in schools: a randomized controlled trial. *Pediatr Infect Dis J* 2011; 30:921–926. 21691245 10.1097/INF.0b013e3182218656 PMC3470868

[18] Larson EL Ferng YH Wong-McLoughlin J Impact of non-pharmaceutical interventions on URIs and influenza in crowded, urban households. *Public Health Rep* 2010; 125:178–191. 20297744 10.1177/003335491012500206 PMC2821845

[19] Cowling BJ Chan KH Fang VJ Facemasks and hand hygiene to prevent influenza transmission in households: a randomized trial. *Ann Intern Med* 2009; 151:437–446. 19652172 10.7326/0003-4819-151-7-200910060-00142

[20] Simmerman JM Suntarattiwong P Levy J Findings from a household randomized controlled trial of hand washing and face masks to reduce influenza transmission in Bangkok, Thailand. *Influenza Other Respir Viruses* 2011; 5:256–267. 21651736 10.1111/j.1750-2659.2011.00205.x PMC4634545

[21] Brankston G Gitterman L Hirji Z Transmission of influenza A in human beings. *Lancet Infect Dis* 2007; 7:257–265. 17376383 10.1016/S1473-3099(07)70029-4

[22] Boone SA Gerba CP The occurrence of influenza A virus on household and day care center fomites. *J Infect* 2005; 51:103–109. 16038759 10.1016/j.jinf.2004.09.011

[23] Simmerman JM Suntarattiwong P Levy J Influenza virus contamination of common household surfaces during the 2009 influenza A (H1N1) pandemic in Bangkok, Thailand: implications for contact transmission. *Clin Infect Dis* 2010; 51:1053–1061. 20879867 10.1086/656581

[24] Lee T Jordan NN Sanchez JL Selected nonvaccine interventions to prevent infectious acute respiratory disease. *Am J Prev Med* 2005; 28:305–316. 15766621 10.1016/j.amepre.2004.12.010 PMC7135187

